# Porcine model of neurocysticercosis by intracarotid injection of *Taenia solium* oncospheres: Dose assessment, infection outcomes and serological responses

**DOI:** 10.1371/journal.pntd.0010449

**Published:** 2022-06-02

**Authors:** Gianfranco Arroyo, Luz Toribio, Ana Vargas-Calla, Juan F. Calcina, Edson Bernal, Nancy Chile, Miguel Zambrano, Luis A. Gomez-Puerta, Juan Chacaltana, Miguel Marzal, Javier A. Bustos, Manuela R. Verastegui, Robert H. Gilman, Seth E. O’Neal, Armando E. Gonzalez, Hector H. Garcia, for The Cysticercosis Working Group in Peru

**Affiliations:** 1 Center for Global Health, Universidad Peruana Cayetano Heredia, Lima, Peru; 2 Cysticercosis Unit, Instituto Nacional de Ciencias Neurologicas, Lima, Peru; 3 Faculty of Veterinary Medicine, Universidad Nacional Mayor de San Marcos, Lima, Peru; 4 Infectious Diseases Laboratory Research-LID, Faculty of Sciences and Philosophy, Universidad Peruana Cayetano Heredia, Lima, Peru; 5 Department of Imaging Diagnosis, Instituto Nacional de Ciencias Neurologicas de Lima, Lima, Peru; 6 School of Medical Technology, Faculty of Medicine, Universidad Peruana Cayetano Heredia, Lima, Peru; 7 Department of International Health, Bloomberg School of Public Health, Johns Hopkins University, Baltimore, Maryland, United States of America; 8 School of Public Health, Oregon Health & Sciences University-Portland State University, Portland, Oregon, United States of America; Universidad de la Republica, Uruguay, URUGUAY

## Abstract

**Background:**

Neurocysticercosis (NCC) is the infection of the human central nervous system (CNS) by *Taenia solium* larvae that cause significant neurological morbidity. Studies on NCC pathophysiology, host-parasite interactions or therapeutic agents are limited by the lack of suitable animal models. We have previously reported that carotid injection of activated *T*. *solium* oncospheres directs parasites into the CNS and consistently reproduces NCC. This study assessed the minimal dose required to consistently obtain NCC by intracarotid oncosphere injection and compared antigen and antibody response profiles by dose-group.

**Methods/Principal findings:**

Three groups of pigs were infected with either 2500 (n = 10), 5000 (n = 11), or 10000 (n = 10) oncospheres. Two pigs died during the study. Necropsy exam at day 150 post-infection (PI) demonstrated viable NCC in 21/29 pigs (72.4%), with higher NCC rates with increasing oncosphere doses (4/9 [44.4%], 9/11 [81.8%] and 8/9 [88.9%] for 2500, 5000, and 10000 oncospheres respectively, *P* for trend = 0.035). CNS cyst burden was also higher in pigs with increasing doses (*P* for trend = 0.008). Viable and degenerated muscle cysticerci were also found in all pigs, with degenerated cysticerci more frequent in the 2500 oncosphere dose-group. All pigs were positive for circulating parasite antigens on ELISA (Ag-ELISA) from day 14 PI; circulating antigens markedly increased at day 30 PI and remained high with plateau levels in pigs infected with either 5000 or 10000 oncospheres, but not in pigs infected with 2500 oncospheres. Specific antibodies appeared at day 30 PI and were not different between dose-groups.

**Conclusion/Significance:**

Intracarotid injection of 5000 or more oncospheres produces high NCC rates in pigs with CNS cyst burdens like those usually found in human NCC, making this model appropriate for studies on the pathogenesis of NCC and the effects of antiparasitic treatment.

## Author summary

Neurocysticercosis (NCC) is the infection of the central nervous system (CNS) with *Taenia solium* larvae and is considered a neglected disease that causes significant neurological morbidity in humans. Animal models are essential to better understand the pathophysiological processes that occur during NCC infection, disease progression and to evaluate the effects of antiparasitic treatment. Here, we demonstrated that by injecting 5000 or more activated *T*. *solium* oncospheres into the carotid artery of pigs we consistently reproduced NCC in a number that closely resemble human infection. All cysticerci found in the CNS were apparently viable, whereas in the musculature viable and degenerated cysticerci were found. Serum antigens in experimentally infected pigs were detected as early as 14 days post–infection and remained high in NCC pigs until necropsy. Histological analysis of brain cysticerci showed different degrees of inflammatory cell infiltration adjacent to the cyst wall, mostly composed of eosinophils, lymphocytes, and macrophages, whereas most of the cysticerci were apparently intact with cyst wall and neck without damage. Our findings demonstrated that the intracarotid pig model is suitable for studies on the pathogenesis and antiparasitic treatment in NCC.

## Introduction

The pork tapeworm *Taenia solium* causes taeniasis and cysticercosis, a zoonotic disease-complex that is endemic in rural areas of many low-income and middle-income countries where poor sanitation, human open-air defecation, and free-range pig rearing practices are common [1–3]. In the lifecycle of *T*. *solium*, humans are the only definitive host harboring the adult form (tapeworm) in their intestines, whereas pigs are intermediate hosts harboring the tissue larval form (cysticercus) in organs such as muscles, central nervous system (CNS), and eyes, among others [2]. Humans can also acquire cysticercosis infection and become accidental intermediate hosts. The establishment of cysticerci in the CNS this leads to a condition called neurocysticercosis (NCC). NCC is considered the leading cause of preventable epilepsy in most of the world [4,5], and therefore contributes significantly to the burden of epilepsy and other neurological morbidity. NCC affects between 2.5 to 8.3 million people annually, and accounts for an estimate of 2.5 disability-adjusted life years (DALYs) [6]. Despite its importance in global public health, NCC is still considered a neglected tropical disease [1,7].

The study of human NCC is commonly performed in symptomatic patients using neuroimaging techniques such as brain magnetic resonance imaging (MRI) and computed tomography (CT) scan, which allow correlation of neuroimaging findings (developmental stage and location of CNS cysticerci) with clinical manifestations of patients [2,8,9]. Serology provides information about immune responses and the presence of viable and nonviable CNS infection, which complements neuroimaging for a proper diagnosis [10–13]. Less frequently, histopathological and immunohistochemical studies on human NCC using brain biopsies have been conducted, giving new insights about the mechanisms of cyst degeneration, inflammation, blood-brain-barrier (BBB) disruption surrounding cysticerci, perilesional gliosis, and axonal damage [14–17]. However, major disadvantages of studying NCC in humans include the lack of control of variables that determine the onset of the disease (as it can only be studied when it occurs naturally), and the scarcity of brain specimens for histological studies, which limit exploration of processes associated with infection and disease progression in NCC [18].

Animal models for NCC allow better comprehension of the infection course, host–parasite interactions, and immunopathogenesis, and provide an ideal tool for initial testing of new therapies [18,19]. A variety of studies have been conducted using rodents [20–25] and pigs [26–31] as NCC models, each with their own advantages and disadvantages. The use of rodents as NCC models has logistical and economic advantages over large animals such as pigs [19]. However, most studies in rodents use intraperitoneal or intracranial routes for infection [20,21,23–25] that are not part of the natural lifecycle of *T*. *solium*, and which may potentially exacerbate an inflammatory reaction in the CNS, making these models unsuitable for translational studies in humans. On the other hand, the pig is the natural intermediate host of *T*. *solium*, and the characteristics of porcine CNS infection resemble human infection more closely. Although experimental oral infection in pigs reproduces CNS cyst infection [27,28,30], this requires a high dose-inoculum and lacks reproducibility. Naturally infected pigs may overcome this problem, although major drawback of this model derives from the uncertainty about cyst longevity, pre-existing inflammation, and the extremely variable parasite load in the brain of NCC pigs [18,19], factors that complicate comparisons between groups in pre-clinical studies. Surgical implantation of activated oncospheres in the brain of pigs has also been tested [31]; however, a significant percentage of CNS cysticerci are found in degenerative stage, a major disadvantage of this model.

We have previously demonstrated that by injecting activated *T*. *solium* oncospheres in the common carotid artery of piglets it is possible to direct the inoculum to the CNS [32]. This novel pig model consistently reproduces CNS cyst infection and may provide a more feasible alternative for the study of NCC. The present study assessed the reproducibility of our carotid oncosphere-injection model of NCC and determined the optimal dose of oncospheres required to consistently achieve CNS cyst infection. Taking advantage of this novel NCC model, we also described the dynamics of antigens and antibodies during infection, as an attempt to link the serological responses with infection outcomes.

## Materials and methods

### Ethics statement

This study was reviewed and approved by Institutional Ethics Committee for Animal Care and Use of the Universidad Peruana Cayetano Heredia (assurance number 023-04-19). All the study procedures described in this study were in accordance with the guidelines of The Office of Laboratory Animal Welfare (OLAW)–National Institutes of Health (NIH).

### Study design and animals

The study compared infection efficacy, as well as antigen and antibody dynamics, in three experimental pig groups injected with either 2500, 5000 or 10000 activated *T*. *solium* oncospheres into the common carotid artery by surgical catheterization. Briefly, 31 two-month old, mixed-breed, male and female pigs were acquired from a cysticercosis-free farm located in Lima, Peru and transported to our pathogen-free experimental veterinary facilities at San Marcos University in Lima, Peru. Negative results to antibodies in the enzyme-linked immunoelectrotransfer blot (EITB) assay [33,34] and to antigens in an antigen-ELISA assay using anti-*T*. *solium* monoclonal antibodies (McAbs) were considered to rule out previous exposure or recent infection with to *T*. *solium*. Pigs were kept under experimental conditions, housed in groups of five pigs per cage, with 12/12-hour light-dark cycles, and with an average temperature of 21°C. All pigs were also vaccinated against hog cholera and received food and water *ad libitum*.

### Sample size and allocation of pigs in experimental groups

Ten pigs were included in each group. This sample size provided 80% statistical power at 5% significance level to detect a difference of 60 percentage points of NCC infection achieved between the groups infected with the highest and lowest doses of oncospheres (90% versus 30%, respectively). An additional available pig was included in the 5000 oncosphere dose-group (N total = 31 pigs). Experimental infections were carried out in sequential rounds according to the availability of collected tapeworms. In each round of infection, similar numbers of pigs were randomly assigned to experimental groups according to weight.

### Experimental details

Parasites, species differentiation, oncosphere collection, and inoculum preparation

Adult tapeworms were obtained from tapeworm carriers treated in clinical settings with a single oral dose of 2 g. of niclosamide [35,36]. Specimens were stored in falcon tubes (15 mL) containing transport medium (0.25 μg / mL amphotericin B, 100 μg / mL streptomycin, 10 IU / mL penicillin adjusted to a volume of 10 mL of saline solution) at 4°C until use. Differential diagnosis of *T*. *solium* and *T*. *saginata* was performed by microscopic evaluation to distinguish the morphological characteristics of the scolex (presence of a rostellum with hooks in *T*. *solium*) and the gravid proglottids (numbers of uterine branches) [37] and confirmed by amplification and sequencing of the partial COX-1 gene [38,39].

Mature eggs were obtained from gravid proglottids by gentle homogenization with 2.5% dichromate solution (Sigma St. Louis, Missouri), and washed three times with distilled water with centrifugation steps at 2500 *g* x 5 minutes between washes. In vitro hatching of oncospheres was achieved by exposing the eggs to 0.75% sodium hypochlorite solution for 10 minutes at 4°C as described above [40,41]. Oncospheres were then washed three times in RPMI 1640 medium (Sigma St. Louis, Missouri), and activated by incubation with artificial intestinal fluid (0.1 mL of porcine bile, 2 μg / mL of NaHCO3, and 10 μg / mL of pancreatin adjusted to a volume of 100 mL with RPMI medium) for 1 hour at 37°C. After activation, the oncosphere were washed three times in RPMI medium, and the viability was assessed using Trypan Blue staining at 4% in a representative sample of 100 oncospheres [42]. The oncospheres were counted using a Neubauer chamber and separated into individual dose inoculums of 2500, 5000, and 10000 oncospheres in 1 mL of saline solution at 37°C ready to use in an immediate period no longer than 2 hours.

### Infection procedure

Pigs were anesthetized using a combined intramuscular dose of ketamine (30 mg / kg) plus xylacine (2 mg / kg) and maintained for 30 minutes by continuous intravenous infusion of ketamine (5 mg / kg) and fentanyl (0.03 mg / kg) every 15 minutes. Pigs were placed in the supine position, and the ventral neck section was sterilized with 10% povidone-iodine solution. An incision of approximately 2 cm was made in the skin of the neck in the region corresponding to the location of the right common carotid artery. The skin, subcutaneous tissue and sternocleidomastoid muscle were separated to expose the common carotid artery for catheterization using a 22-ga, 10 cm central venous catheterization set (Arrow International, Teleflex Int, Reading, PA). The inocula containing the oncospheres were re-suspended in 10 mL of saline solution and injected through the catheter into the vasculature. A second flush (10 mL) of saline was injected to ensure removal of the oncospheres from the catheter. Immediately afterwards, the incision was sutured, and all pigs received 3-day post-operatory treatment with antibiotics (penicillin 20,000 IU / kg IM) and anti-inflammatory drugs (ketoprofen 2 mg / kg IM).

### Clinical and serological monitoring

Pigs were kept in our veterinary facilities for 150 days and were monitored every day by the veterinary staff for clinical signs. Blood samples (8 mL) were also taken from the cava vein of pigs every week post-infection (PI). Samples were centrifuged at 2500 RPM for 10 minutes to obtain serum aliquots. Sera were evaluated for the presence of antibodies by EITB assay [33,34] and circulating antigens by Ag-ELISA [43].

### Enzyme-linked Immunoelectrotransfer Blot (EITB)

The EITB assay uses seven lentil-lectin purified *T*. *solium* glycoprotein antigens (GP50, GP42-39, GP24, GP21, GP18, GP14 and GP13) obtained by separation in sodium dodecyl sulphate–polyacrylamide gel electrophoresis, transferred to nitrocellulose membranes and incubated in duplicate with pre-treated sera as previously described [33,34]. Antibody responses were expressed as the number of visualized bands with 1 or more bands considered as positive.

### Ag-ELISA

We performed an in-house capture Ag-ELISA assay for the detection of circulating antigens using the set of monoclonal antibodies (McAbs) TsW8/TsW5 produced against *T*. *solium* whole cysticerci components developed by our group [43]. Briefly, ELISA plates were sensitized with the TsW8 McAb (2 μg / mL) diluted in bicarbonate buffer and incubated for 1 hour at 37°C. Plates were then washed five times with 0.05% PBS-Tween and incubated for 30 minutes with a blocking solution. The content was discarded and 100 μL / well of sera pre-treated with 5% trichloroacetic acid (TCA) was added, incubated for 20 minutes at room temperature, and washed. Then, 100 mL of biotinylated McAb TsW5 (2 μg / mL) were added in each well. After another incubation and washing step, streptavidin-HRP diluted at 1:10000 was added and kept in incubation. Finally, 100 μL / well of O-phenylenediamine (OPD) diluted in citrate buffer were added. The enzymatic reaction was stopped with HsSO4, and plates were read in an ELISA reader at 490 / 650 nm of wavelength. Optical density (OD) values were obtained and divided by the OD values from a pool of known negative samples for the calculation of antigen ratios. Samples were positive if antigen ratios were equal or higher than 1.

### Brain magnetic resonance imaging (MRI)

All pigs underwent a brain MRI exam on day 150 PI to identify cysticerci in the CNS. The MRI exam was performed at the Instituto Nacional de Ciencias Neurologicas in Lima, using a 3-Tesla scanner (Phillips, Achieva, Best, The Netherlands); imaging protocols included axial, fluid-attenuated inversion recovery (FLAIR), and Fast imaging employing steady-state acquisition (FIESTA) sequences at 3 mm of thick slice without gap. Vesicular cysticerci appeared on brain MRI as small, well-delimited, rounded low-intensity areas showing in its interior a hyperintense nodule that represents the invaginated scolex [2].

### Necropsy and assessment of cyst infection

After MRI examination, all pigs were euthanized to determine cyst burden in each brain and the entire carcass. For euthanasia, pigs were anesthetized using a combined intramuscular dose of ketamine (30 mg / kg) plus xylacine (2 mg / kg), and then received an intravenous overdose of pentobarbital sodium (120 mg / kg). Euthanasia was confirmed by the absence of pulse and heartbeats on auscultation. Immediately after euthanasia, the brain was perfused with approximately 2000 mL of a chilled solution containing heparin (NaCl 0.85% with heparin 10 IU / mL) by catheterization of the common carotid artery connected to a peristaltic pump. After perfusion, the brain was removed from the skull, kept on dry ice for 15 minutes, and cut into 10 mm coronal sections to expose the brain parenchyma. Brain cysticerci were macroscopically identified and registered according to brain hemisphere. Cyst anatomical location was determined on histology. A systematic inspection of the entire carcass was also performed by tissue slices of approximately 0.5 cm or less in the musculature using a surgical blade. Cysticerci recovered were classified according to their macroscopic characteristics as vesicular (cysticerci with clear vesicular fluid, well-delineated, thin-walled cyst structures, and a visible invaginated scolex), or degenerated (dense cyst structures with caseous content, without fluid nor scolex visible). The cyst burden of vesicular and degenerated cysticerci in the CNS and the musculature was recorded for each pig.

### Cyst evagination test

*In vitro* evagination test using whole bovine bile was performed on a subsample of vesicular cysticerci from each pig to determine the proportion of vesicular cysticerci that were viable and capable of developing into an adult tapeworm [29,32]. Cyst viability was assessed by the presence of a moving scolex outside the cyst wall.

### Histology

A subsample of CNS cyst specimens with adjacent brain tissue were collected shortly after necropsy and fixed in 10% neutral buffered formalin (37% formaldehyde in phosphate buffered solution, pH 7.2) for 24 hours, and paraffin-embedded for sectioning. Paraffin-embedded serial sections (4 μm in thickness) were mounted on slides for histological analysis of cyst tissue and inflammatory cell infiltrate using standard hematoxylin and eosin (HE) stain methods.

### Statistical analysis

NCC rates were calculated by dividing the number of pigs infected with CNS cysticerci by the total number of pigs in each dose group. The gradient of NCC rates according to dose group was assessed using the nonparametric Cochran-Armitage test for trend; NCC rates between dose groups were also compared using Fisher’s exact test. The percentages of infection efficiency with total cysticerci (vesicular + degenerated) and vesicular cysticerci only (obtained by dividing the number of total and vesicular cysticerci by the number of oncospheres administered in each group and multiplied by 100) were also compared by dose groups using Fisher’s exact test. Cyst burden (vesicular and degenerated cysticerci) found in the CNS and the musculature of pigs were summarized using medians with interquartile ranges (IQR) according to dose groups, assessed for trend using the nonparametric Cuzick test, and compared between dose groups using the nonparametric one-way ANOVA (Kruskal Wallis test). Percentages of CNS cysticerci distributed according to brain hemisphere and CNS anatomical location were also described. The dynamics of antigens (OD ratios) and antibodies (number of EITB bands) at follow up (days 0, 7, 14, 30, 50, 70, 100, 130, and 150 PI respectively) were summarized using means ± standard errors and medians with IQR by dose group. Differences in antigen levels over time between dose groups were assessed using random-effects linear regression model, and differences in the numbers of antibody bands were assessed using nonparametric regression models. The overall correlations between the number of vesicular cysticerci and total cysticerci (vesicular + degenerated) found during necropsy with antigen and antibody levels were performed using nonparametric Spearman’s rank correlation coefficients. Statistical analysis was carried out in Stata/SE 17.0 (Stata Corp., College Station, TX), and plot were created using the ggplot package in RStudio V1.4.1106. *P* values under 0.05 were statistically significant.

## Results

A total of five *T*. *solium* tapeworms confirmed by microscopic exam and PCR assay were used for experimental infections in pigs at different times. Five rounds of infection were carried out using a single tapeworm in each round and infecting two pigs per experimental group (except in the 5000 oncosphere-dose group where 3 pigs were infected in the last round). Percentages of oncosphere activation ranged from 40% to 80% (average of 51.4%, Table A in S1 Tables).

Two pigs were euthanized during the study due to gastric torsion and severe pneumonia, respectively. The remaining 29 pigs completed clinical and serological follow up until necropsy at day 150 PI. None of these pigs showed any neurological signs nor signs of discomfort throughout the entire study period.

**Fig 1:**
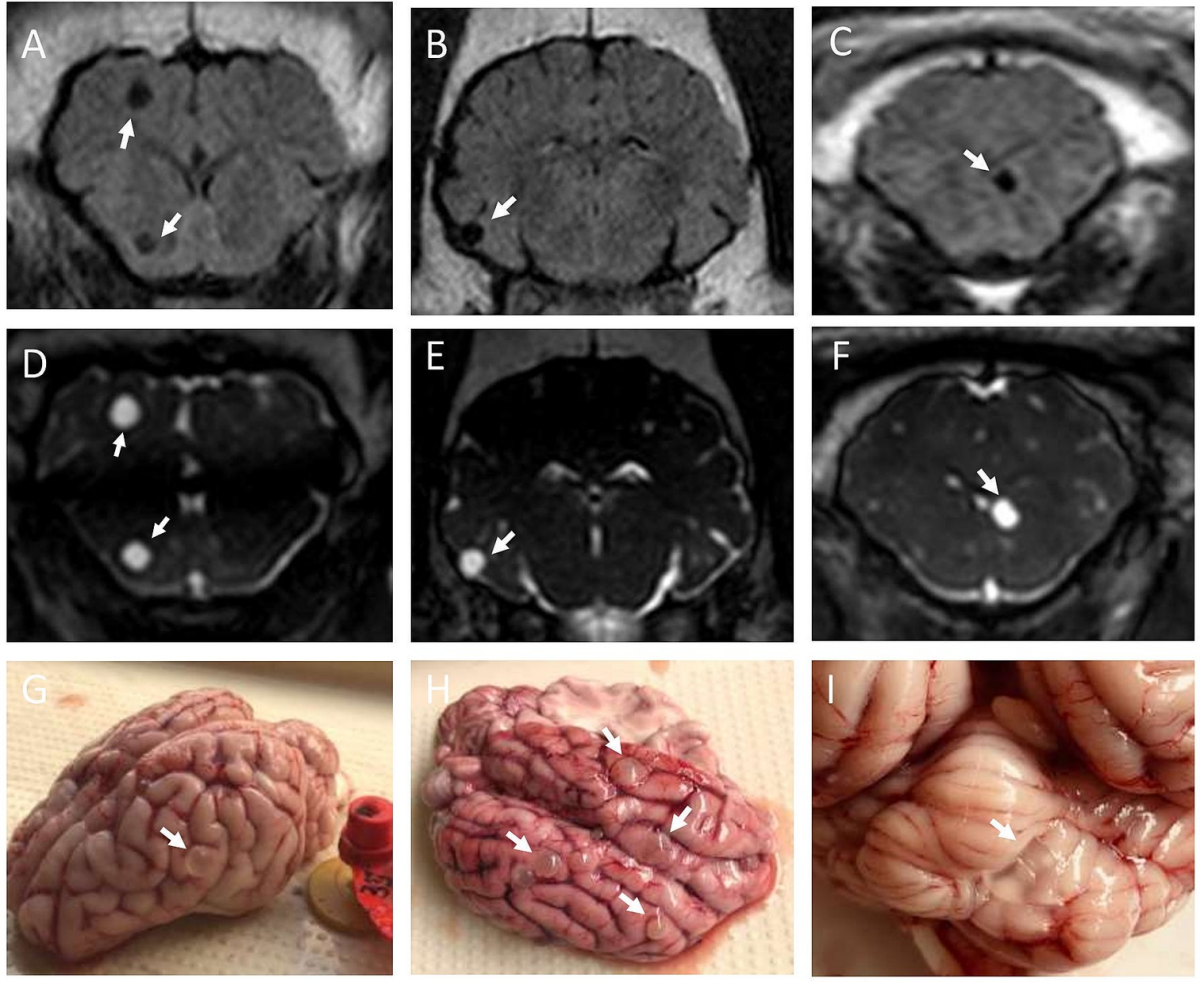
Brain MRI findings and gross examination of pigs’ brains showing cysticerci (white arrows). (A-C) Fluid-attenuated inversion recovery (FLAIR) sequences; (D-F) Fast imaging employing steady-state acquisition (FIESTA) sequences; (G-H) Corticomeningeal cysticerci; (I) Cysticerci lodged in the cerebellum.

Brain MRI exam at day 150 PI demonstrated CNS cyst infection in 21 out of 29 pigs (72.4%), which were later confirmed by necropsy exam. All CNS cysticerci were vesicular (as visualized on brain MRI and gross evaluation, Fig 1) and no degenerated cysticerci were found in the pig’s brains. We observed a trend towards higher rates of NCC with increasing dose groups (*P* for trend = 0.035, Table 1). Higher rates of NCC were found in pigs infected with 5000 and 10000 oncospheres (81.8% [9/11] and 88.9% [8/9] respectively) compared to pigs infected with 2500 oncospheres (44.4% [4/9], *P* = 0.134). All pigs with NCC also developed cysticercosis in their muscles (either vesicular or degenerated cysticerci). In addition, a pig in the 2500 oncosphere dose-group harbored only degenerated cysticerci in the musculature but was negative for NCC.

**Table 1:** Distribution of pigs with cysticercosis and NCC, as well as efficiency of infection (%) according to oncosphere dose–groups.

Oncosphere dose	N	Cysticercosis^a^		NCC^b^		Efficiency of infection with total cysticerci (vesicular or degenerated)		Efficiency of infection with vesicular cysticerci	
		n (%)	*P*	n (%)	*P*	C/O^c^ (%)	*P*	C/O^c^ (%)	*P*
2500	9	9 (100.0)	–	4 (44.4)	0.134	337/22500 (1.5)	< 0.001	137/22500 (0.6)	< 0.001
5000	11	11 (100.0)		9 (81.8)		1402/55000 (2.6)		1279/55000 (2.3)	
10000	9	9 (100.0)		8 (88.9)		1947/90000 (2.2)		1777/90000 (1.9)	
Total	29	29 (100.0)		21 (72.4)		3686/167500 (2.2)		3193/167500 (1.9)	

^a^Presence of either vesicular or degenerated cysticerci in the musculature of pigs

^b^Presence of vesicular cysticerci in the pig’s brains

^c^Number of cysticerci / number of oncospheres

A total of 337, 1402, and 1947 total cysticerci (either vesicular or degenerated) were recovered from pigs infected with 2500, 5000, and 10000 oncospheres, of which, 137, 1279, and 1777 were vesicular cysticerci. Infection efficiency with total cysticerci was higher with increasing dose groups (1.5%, 2.6%, and 2.2% for 2500, 5000, and 10000 oncospheres, *P* < 0.001), and a similar effect was observed for infection efficiency with vesicular cysticerci (0.6%, 2.3%, and 1.9% for 2500, 5000, and 10000 oncospheres respectively, *P* < 0.001 Table 1).

**Fig 2:**
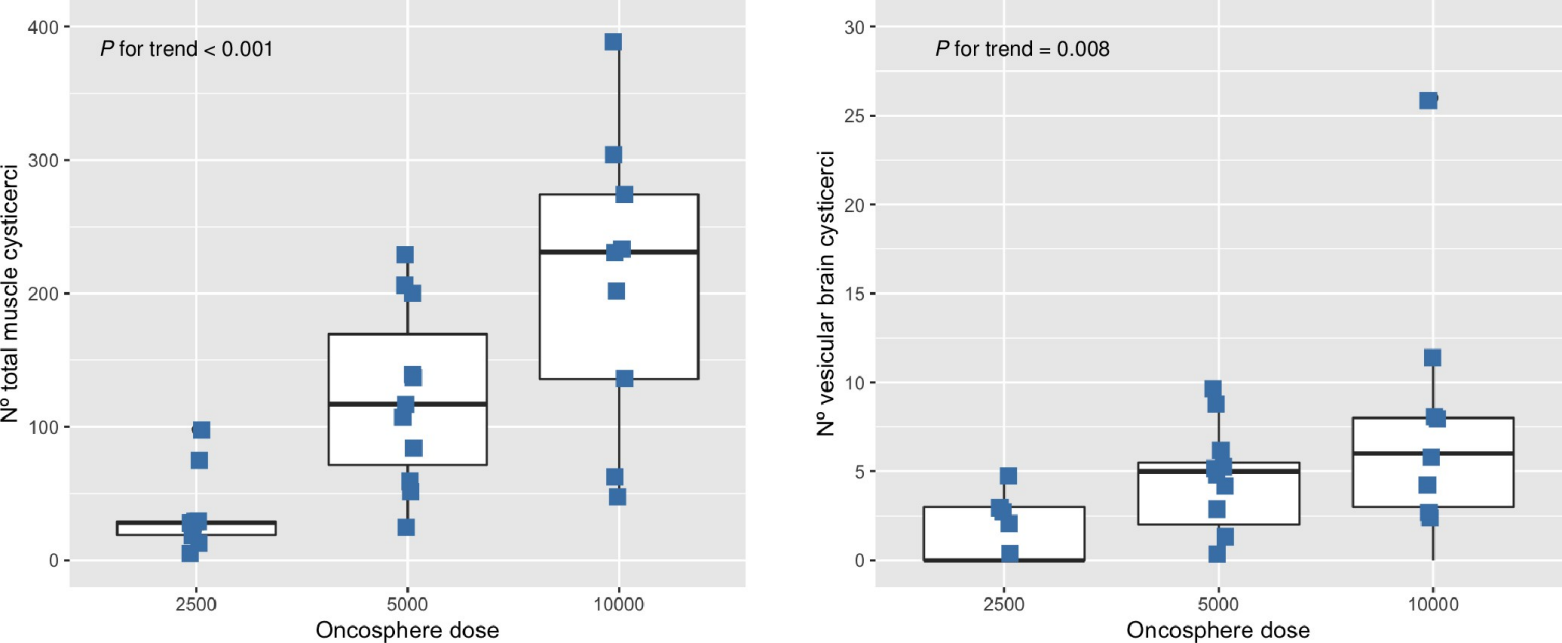
Boxplots showing the distribution of total muscle cysticerci (A) and vesicular CNS cysticerci according to dose groups. P value was obtained by using nonparametric Cuzick’s trend test. The median is represented as the horizontal line in the box, and the lines (whiskers) outside the wall are extended to cover all data points that lie within 1.5 ± IQR (interquartile ranges) from upper and lower quartiles respectively. Data points outside whiskers represent outliers.

A total of 129 CNS vesicular cysticerci were found in all NCC pigs at the time of necropsy. CNS cysticerci represented less than 4% of total cysticerci recovered from pigs in all groups (3.9% [13/337], 3.4% [48/1402], and 3.5% [68/1947] for 2500, 5000, and 10000 oncospheres respectively, *P* = 0.902, Table 2). We also observed a gradient of CNS loads with vesicular cysticerci with increasing dose groups (*P* for trend = 0.008, Fig 2). Higher loads with CNS cysticerci were found in pigs infected with 5000 and 10000 oncospheres (median: 5 cysticerci [IQR: 1–6] and median: 6 cysticerci [3–8]) compared to pigs infected 2500 oncospheres (median: 0 cysticerci [IQR: 0–3], *P* = 0.025). On the other hand, a total of 3064 vesicular cysticerci were found in the musculature of pigs. Vesicular muscle cysticerci composed more than 80% of total cysticerci found in pigs infected with 5000 and 10000 oncospheres (87.8% [1231/1402], and 87.8% [1709/1947] respectively, Table 2), but less than 40% of total cysticerci in pigs infected with 2500 oncospheres (36.8% [124/337], *P* < 0.001). Parasitic loads with vesicular cysticerci in the musculature were also higher in increased oncosphere dose groups (median: 111 cysticerci [IQR: 54–141] for 5000 oncospheres, and median: 190 cysticerci [IQR: 136–233] for 10000 oncospheres, compared to median: 6 cysticerci [IQR: 2–18] for 2500 oncospheres, *P* < 0.001). Parasitic loads with degenerated muscle cysticerci were higher in pigs infected with 2500 oncospheres (median: 16 cysticerci [IQR: 3–26]) compared to pigs infected with 5000 and 10000 oncospheres (median: 0 cysticerci [IQR: 0–5] and median: 8 cysticerci [IQR: 2–12], *P* = 0.121). Cyst evagination test was performed in 485 vesicular cysticerci (459 collected from muscle and 26 collected from brains) We observed higher percentages of viability with increasing oncosphere doses (75.5%, 92.5%, and 94.6% for 2500, 5000 and 10000 oncospheres respectively, *P* = 0.001, Table 2).

**Table 2:** Necropsy results (cyst burden with either vesicular or degenerated cysticerci in muscle and CNS) and percentages of cyst viability in pigs according to oncosphere dose-groups.

Oncosphere dose	Pig Number	Total cysticerci*	Muscle cysticerci		CNS cysticerci		Cyst viability (%)
			Vesicular	Degenerated	Vesicular	Degenerated	
2500							
	1 2 3 4 5 6 7 8 9	31 29 28 13 5 101 21 29 80	6 13 2 0 2 5 18 29 49	22 16 26 13 3 93 1 0 26	3 0 0 0 0 3 2 0 5	0 0 0 0 0 0 0 0 0	4/6 (66.7) 2/4 (50.0) 0/2 (0.0) - 2/2 (100.0) 0/2 (0.0) 10/10 (100.0) 11/13 (84.6) 8/10 (80.0)
	Total Median^†^ % Total	337 29 (21–31) -	124 6 (2–18) 36.8	200 16 (3–26) 59.4	13 0 (0–3) 3.9	0 - 0.0	37/49 (75.5)
5000							
	10 11 12 13 14 15 16 17 18 19 20	59 89 205 25 238 210 142 118 142 57 117	54 80 177 25 141 206 139 114 137 51 107	5 4 23 0 88 0 0 3 0 0 0	0 5 5 0 9 4 3 1 5 6 10	0 0 0 0 0 0 0 0 0 0 0	18/22 (81.8) 25/28 (89.3) 26/26 (100.0) 10/11 (90.9) 39/40 (97.5) 23/25 (92.0) 17/20 (85.0) 18/20 (90.0) 23/25 (92.0) 10/10 (100.0) 24/25 (96.0)
	Total Median^†^ % Total	1402 118 (59–205) -	1231 114 (54–141) 87.8	123 0 (0–5) 8.8	48 5 (1–6) 3.4	0 - 0.0	233/252 (92.5)
10000							
	21 22 23 24 25 26 27 28 29	400 210 142 237 233 274 51 70 330	381 190 136 233 225 163 46 40 295	8 12 0 0 6 111 2 22 9	11 8 6 4 2 0 3 8 26	0 0 0 0 0 0 0 0 0	18/18 (100.0) 17/20 (85.0) 20/20 (100.0) 26/26 (100.0) 20/20 (100.0) 17/19 (89.5) 21/21 (100.0) 18/20 (90.0) 17/20 (85.0)
	Total Median^†^ % Total	1947 233 (142–274) -	1709 190 (136–233) 87.8	170 8 (2–12) 8.7	68 6 (3–8) 3.5	0 - 0.0	174/184 (94.6)

^†^Interquartile range

CNS cysticerci were distributed in both hemispheres (43.4% [56/129] in left and 53.5% [69/129] in right); three cysticerci were contained within the midline and one cysticercus was found in the cerebellum. Although 129 cysticerci were recovered from the pig brains at the time of necropsy, cyst anatomical location was assessed only in 103 cysticerci preserved in formalin (26 cysticerci were not included because were used for cyst viability test and could not be fixed in formalin for further analysis). Most cysticerci on histology were classified as corticomeningeal (50.5% [52/103]) and parenchymal (41.8% [43/103], whereas only 7 (6.8%) were considered meningeal (see Table B in S1 Tables). On the other hand, cysticerci found in the musculature of pigs in all dose groups were mainly distributed in legs, forelegs, and diaphragm (see Table C in S1 Tables).

**Fig 3:**
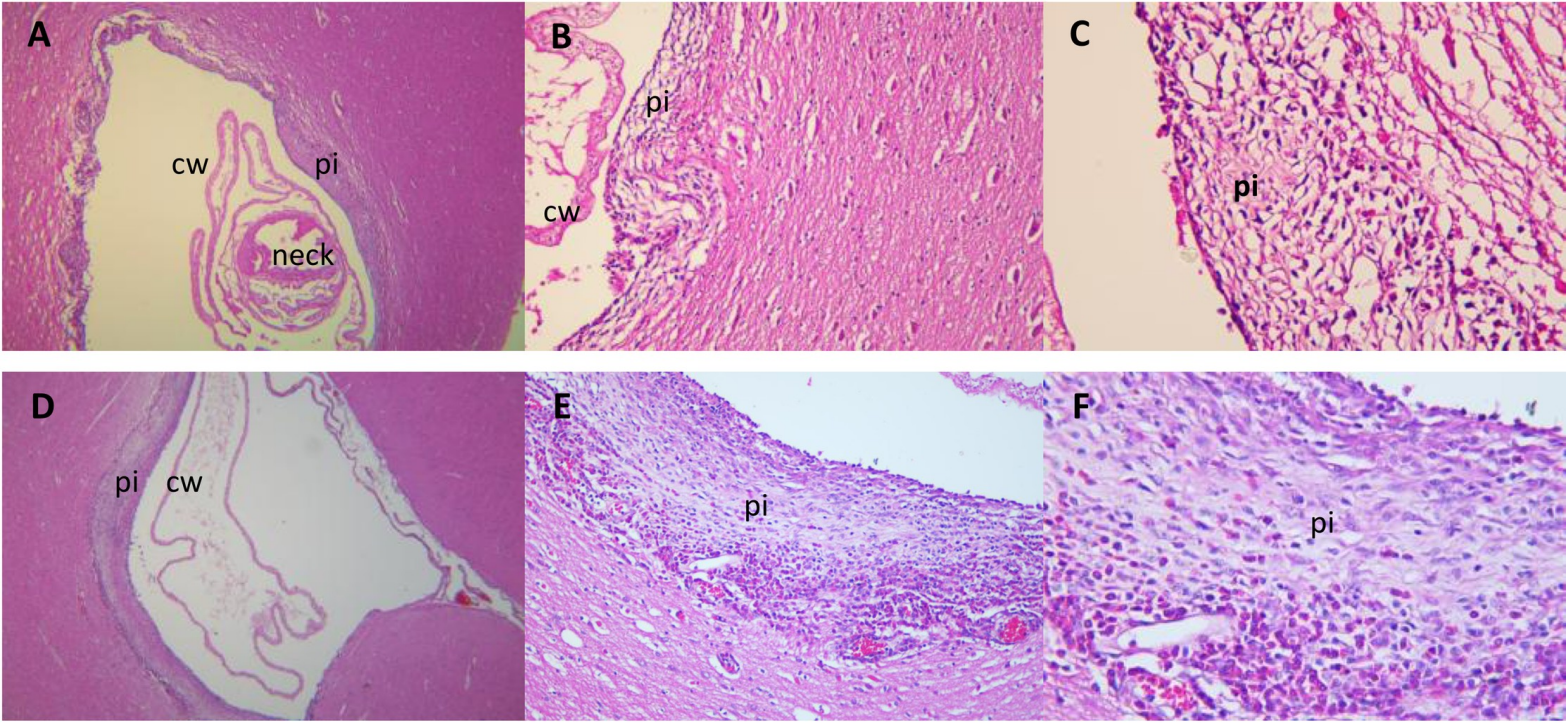
Histopathology of H&E-stained sections of two brain cysticerci with pericystic inflammation recovered from different experimental NCC pigs. (A), (B), and (C) show a parenchymal cyst with adjacent tissue at x4, x20, and x40 magnification respectively. Moderate pericystic inflammation in all areas surrounding the parasite is observed with a moderate number of inflammatory cells (mainly eosinophils and lymphocytes), and cyst wall and neck appears to be intact. (D), (E), and (F) show a corticomeningeal cyst with abundant pericystic inflammation in the cortical parenchyma at x4, x20, and x40 magnification. Three pericystic cell layers were observed, the first corresponded to abundant epithelioid cells, eosinophils, and macrophages dispersed between collagen fibers. The second layer includes numerous eosinophils and macrophages. The third layer included plethoric meningeal vessels with abundant extravasated eosinophils, lymphocytes, and epithelioid cells. Cyst wall appeared with slight damage. Key: cw = cyst wall; pi = pericystic inflammation.

Histological analysis showed brain tissue adjacent to the cyst wall with different inflammatory cell degrees, composed of abundant inflammatory cells, mainly of eosinophils, lymphocytes, macrophages, and epithelioid cells, distributed between the brain tissue and collagen fibers, and certain areas with extravasation of inflammatory cells (Fig 3). On the other hand, most of the cysticerci were apparently intact, with typical cyst wall and neck tissue without damage, or with some areas with slight cellular infiltration.

**Fig 4:**
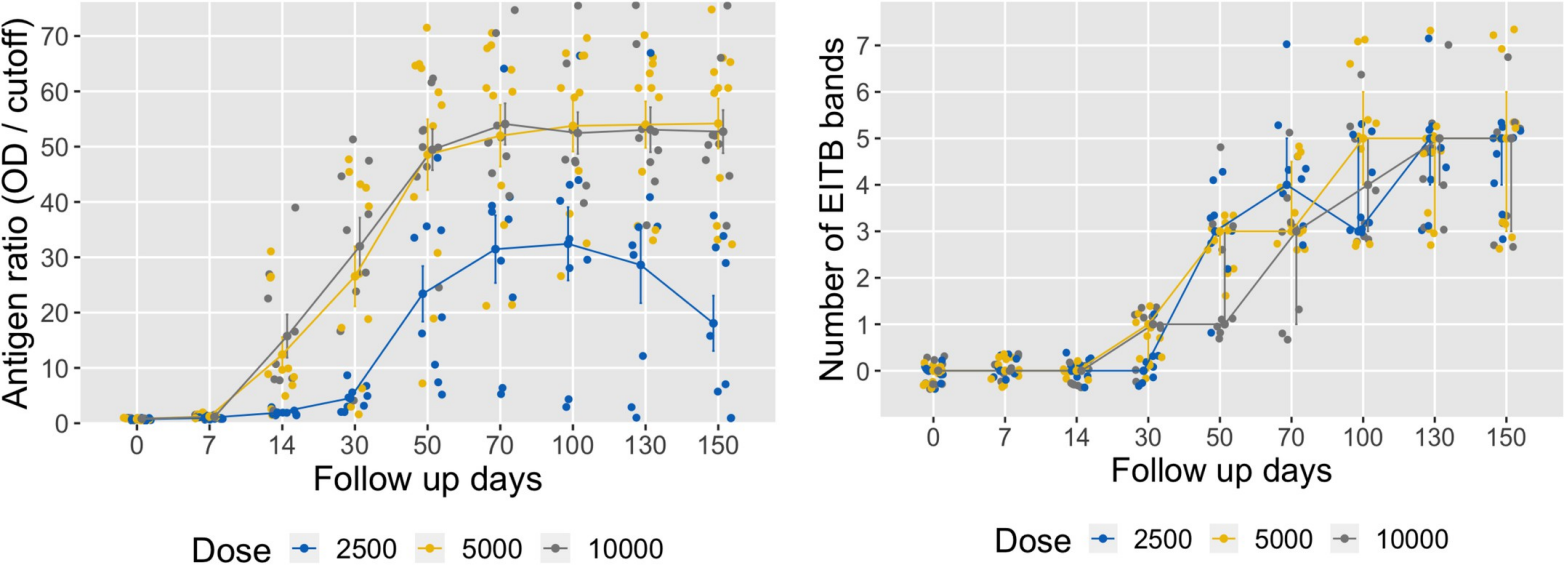
Dynamics of antigen and antibody response profiles at follow up days according to oncosphere-dose groups. (A) Antigen levels summarized using means ± standard errors; (B) Numbers of EITB bands summarized using medians with IQR (interquartile ranges).

There were higher circulating parasite antigen levels in pigs infected with 5000 and 10000 oncospheres versus pigs infected with 2500 oncospheres (*P* < 0.001); antigen response profiles over time in pigs infected with either 5000 or 10000 oncospheres were not different (see coefficients from random-effects regression model in Table D in S1 Tables). At day 7 PI, 16/29 pigs (55.2%) were positive on Ag-ELISA, although circulating antigen levels were low (ratios < 3). At day 14 after infection, all pigs were positive on Ag–ELISA, with a steep increment in serum antigen levels in pigs infected with higher doses of oncospheres (mean antigen ratio: 15.8 ± 3.9 for 10000 oncospheres, and mean antigen ratio: 12.4 ± 3.1 for 5000 oncospheres, versus mean antigen ratio: 1.9 ± 0.2 for 2500 oncospheres). Antigen levels further increased in all dose groups at day 30 PI. Serum antigen levels in pigs infected with 5000 and 10000 remained high with a plateau effect at day 50 PI and onwards until necropsy, whereas antigen levels in pigs infected with 2500 oncospheres reached the highest level at day 100 PI and subsequently declined until necropsy (Fig 4A).

Overall antibody responses on EITB over time were not different between dose groups (Table D in S1 Tables). EITB antibodies appeared in 14/29 pigs (48.3%) at day 30 after infection (GP50 band only). At day 50 PI, all pigs were positive on EITB, with stronger responses seen in pigs infected with 5000 and 2500 oncospheres (median of EITB bands: 3 [IQR: 2–3], versus median of EITB bands: 1 [IQR: 1–3] for pigs infected with 10000 oncospheres, Fig 4B; EITB responses remained high in all pigs at day 70 PI and onwards. At necropsy (day 150 PI) EITB responses were similar in all dose-groups (median of EITB bands: 5).

**Fig 5:**
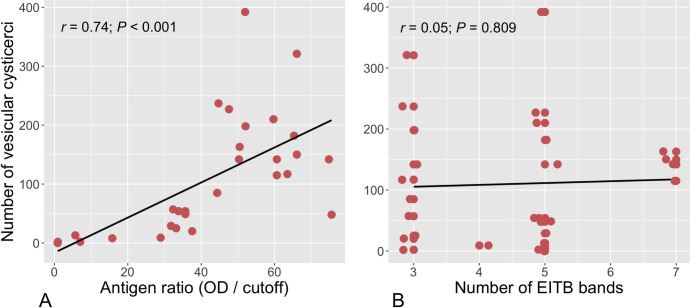
Spearman’s rank correlations coefficients between the number of vesicular cysticerci found in pigs at the time of necropsy with antigen levels on Ag-ELISA (A) and antibody bands on EITB (B).

Antigen levels significantly correlated with parasitic loads of vesicular cysticerci in pigs at the time of necropsy (*r* = 0.74, *P* < 0.001, Fig 5A). On the other hand, no correlation between EITB antibody responses and parasitic loads in pigs was found (*r* = 0.05, *P* = 0.809, Fig 5B). Correlation levels did not vary considerably when considering parasitic loads with total (vesicular + degenerated) cysticerci (*r* = 0.72, *P* < 0.001 for Ag-ELISA, and *r* = 0.22, *P* = 0.251 for EITB antibody bands).

## Discussion

Animal models are essential to understand the pathophysiological mechanisms associated with disease in humans, as well as for diagnostic and pre-clinical therapeutic studies [18]. We had previously demonstrated the development of NCC in pigs by intracarotid injection of activated *T*. *solium* oncospheres [32]. This study consistently reproduced prior findings and showed that a dose of 5000 oncospheres is sufficient to achieve CNS cyst infection in more than 80% of pigs. Parasitic loads in our carotid NCC model (median of 5 cysticerci per pig brain) closely resemble those in human CNS infection, with the additional advantage that all CNS cysts were apparently viable, providing a model suitable for assessing different treatments in pre-clinical therapeutic studies in NCC.

Infections with higher parasitic loads in the CNS and musculature of pigs were obtained by injecting 5000 or 10000 oncospheres versus 2500 oncospheres, demonstrating a dose–dependent phenomenon, as observed in previous experimental studies in pigs [27, 29]. The lower number of total cysticerci (vesicular and degenerated) and the higher percentage of degenerated cysticerci found in the musculature of pigs infected with 2500 oncospheres suggests that low-dose infections may be overcomed by the host’s immunity (probably non-specific immune mechanisms), particularly in the skeletal musculature. This may explain the observation that most naturally infected pigs have low parasite burdens [44, 45]. Other factors such as the age of pigs at the time of infection may also affect the efficiency of infection as previously reported [46].

We chose to make a small surgical incision to expose the common carotid artery for infection procedures. The deep location and position of this vessel in relation to the sternocleidomastoid muscle complicates ultrasound-guided catheterization, which can be associated with hematoma and uncontrolled bleeding in pigs [47], and can also produce accidental extravasation of the inoculum into the musculature of pigs as previously reported [32]. The surgical procedure for infection in pigs did not take more than 30 minutes for the veterinary staff, and we did not report any adverse events in pigs during or immediately after surgery.

MRI findings demonstrated CNS cysticerci in all pigs with NCC confirmed by necropsy. The characteristics of the CNS cysticerci seen in the neuroimages of pigs (more easily in T2–weighted imaging) [48, 49] were very similar to those observed in human NCC, with some cysticerci clearly showing the invaginated scolex [2, 3]. All cysticerci appeared to be viable on MRI and on gross examination. We did not observe pericystic edema on MRI. Brain MRI in the carotid NCC model can be used to randomize pigs with NCC according to parasitic load and cyst characteristics, a major advantage in studies with few animals [19].

All the cysticerci found in the CNS of pigs were apparently viable. These results were consistent with our previous study [32] and similar to previous experimental studies in orally infected NCC pigs [27, 28, 30]. Pigs in our study were not immunosuppressed, and the presence of vesicular cysticerci in the CNS clearly reflects the magnitude of the BBB as a gatekeeper to the brain, as it controls the host’s immune response towards brain cysticerci by regulating the access of immune cells and chemokines into the CNS, which favors the establishment and survival of cysticerci without apparent damage for longer periods [50]. In our model, oncospheres bypass the intestinal mucosa but establish through the natural blood vessel route. This is a major advantage over existing pig or rodent models which rely on direct injection of the inoculum into the brain, a more invasive procedure that may trigger the host’s immune response to the inherent wound and may eventually lead to exacerbated inflammation and cyst degeneration [25, 31]. Although it has been suggested that the passage of oncospheres through the gastrointestinal tract gives them some protection to establish and survive in the CNS [30, 40, 41], we cannot rule out the possibility that faster reach of the parasite to the protected brain tissue by avoiding intestinal exposure to the host’s immune system facilitates the establishment of cysticerci in a less-primed environment.

Oncospheres invaded and developed into vesicular cysticerci in the parenchymal and corticomeningeal regions of the brain, similar to what has been previously observed in the natural pig model of NCC and in human NCC [2, 51]. This suggests that the carotid oncosphere-injection model can be used to study the immunopathological processes according to brain location and its effects in neuroinflammation [52]. The injection of oncospheres through the common carotid artery in our model also produced cysticerci in the musculature. One of the main challenges for the delivery of oncospheres to the CNS in our model is the anatomical arrangement of the porcine cerebral vasculature; the presence of the *rete mirabile* [32, 53] and extensive collateral communications between the internal and external carotid irrigation may explain the delivery of oncospheres to the musculature. Most of the cysticerci found in the musculature of pigs were distributed in legs and forelegs in all doses-groups, similar to previous studies in naturally infected pigs [54], which seems to be related with a greater volume of muscle in those areas for the establishment of cysticerci. Whether the presence of muscle cysticerci may represent a drawback in our model compared to pure CNS infection in intracranial infection models is arguable [18, 19, 25], since serological and cellular responses observed in human NCC may also reflect the effects of extracerebral infections. On the other hand, the inflammatory responses seen in the brain tissue adjacent to vesicular cysticerci in our model were very similar to those observed in naturally infected pigs with NCC, and in human NCC, except for the higher number of eosinophils observed in the brain tissue of pigs, which can reflect an early inflammatory reaction compared to a more chronic response observed in humans [55]. Further studies are needed to characterize the neuroinflammatory reaction seen in our model.

Antigen dynamics in our carotid NCC model were markedly different among pigs infected with either 10000 or 5000 oncospheres compared with pigs infected with the lowest oncosphere dose (2500). Circulating antigens reached detectable levels as early as 7 days PI in 55% pigs, and in 100% of pigs in all dose groups at day 14 PI, similar to previous studies [30, 46]. However, in pigs infected with the higher doses, antigen levels were higher and maintained a plateau at day 50 PI and afterwards. In contrast, pigs infected with the low dose had lower overall levels of antigen, which peaked at day 100 PI then declined until the time of necropsy. Antigen dynamics in pigs infected with 5000 and 10000 oncospheres were very similar despite the presence of higher parasite loads in the latter group, suggesting that the amount of circulating parasite antigens released in pigs in both groups exceeded the detection threshold of our Ag-ELISA assay. Antigen levels in our carotid NCC model were measured using an in-house Ag-ELISA based on our McAbs TsW8/TsW5 (IgM isotypes) that recognize regions of the parasite neck and cyst wall [43]. The presence of transient circulating antigens that later decayed throughout the course of infection in pigs infected with 2500 oncospheres correlates with the amount of degenerating cysticerci found in this group, which reflects the processes leading to cyst degeneration after attack by the immune system as observed previously in studies using oral infection models [27, 46, 56]. The overall correlation between antigen levels and parasitic loads in our study may serve to monitor and quantify the infection process in the carotid pig model of NCC.

On the other hand, antibody responses were not different between oncosphere dose-groups in our model. In our study, all pigs were EITB–positive at day 50 PI, similar to previous experimental studies [31, 46], although in oral ingestion there is an association in the magnitude of the immune response and the infective dose that does not seem to be marked in our model. It is likely that the lack of recognition by the immune system in the intestinal mucosa of the oncospheres injected into the carotid artery may contribute to a higher infection efficiency in the CNS and the musculature in our model. Further studies will be required to determine exact differences in the immune response process between the carotid and the oral model of NCC in pigs.

Some drawbacks in our study deserve to be considered. Our model is not appropriate for studying extraparenchymal NCC (subarachnoid or intraventricular) due to the chronic course of this disease in humans compared to the short lifespan in pigs, and because almost all cysticerci in the CNS of pigs had intraparenchymal or corticomeningeal location. None of the NCC pigs developed neurological signs such as seizures during follow up, as has been shown in rat NCC model [25]. However, the absence of seizures due to NCC in our model does not necessarily exclude the presence of CNS alterations that can be detected and monitored with neuroimaging or electrocorticography. There are also logistic disadvantages related to handling large animals that consume resources, but these need to be balanced with the availability of a more efficient NCC model.

Despite the great impact of NCC on public health and the large number of clinical studies and animal models previously described in the literature, there are still little–known aspects about NCC pathology, the immune and inflammatory mechanisms that occur during the course of infection and after anthelmintic treatment, as well as their relationship with seizure development [18, 19]. Here, we demonstrated that experimental NCC in pigs by carotid oncosphere injection is clearly dose dependent and the CNS is a privileged site for viable infection compared to the musculature. We also determined that using a minimum dose of 5000 oncospheres produces high rates of CNS cyst infection with parasitic loads similar to human NCC. Despite evident differences in clinical manifestations between porcine and human NCC, the intracarotid pig infection may constitute an efficient model for studies tracing the pathogenesis of NCC and the effects of antiparasitic treatment.

## Supporting information

S1 Tables **Table A.** Tapeworms used for infection rounds in pigs. **Table B.** Distribution of cysticerci in the CNS of pigs according to hemisphere and anatomical location. **Table C.** Detailed distribution of cysticerci (vesicular + degenerated) found in the carcasses of pigs according to oncosphere dose. **Table D.** Differences in the dynamics of circulating antigens and antibodies over time in experimentally infected pigs according to oncosphere dose–groups.
(DOCX)
