# Supplementary material for: Porcine model of neurocysticercosis by intracarotid injection of Taenia solium oncospheres: Dose assessment, infection outcomes and serological responses
Source: PLoS Negl Trop Dis. 2022 Jun 2;16(6):e0010449. doi: 10.1371/journal.pntd.0010449 (PMC9162370; doi:10.1371/journal.pntd.0010449)
Supplement: S1 Tables — Table A. Tapeworms used for infection rounds in pigs. Table B. Distribution of cysticerci in the CNS of pigs according to hemisphere and anatomical location. Table C. Detailed distribution of cysticerci (vesicular + degenerated) found in the carcasses of pigs according to oncosphere dose. Table D. Differences in the dynamics of circulating antigens and antibodies over time in experimentally infected pigs according to oncosphere dose–groups. (DOCX) [file pntd.0010449.s001.docx]

**Supplementary information S1**

**Table A.** Tapeworms used for infection rounds in pigs

| Tapeworm code | Patients procedence | Number of proglottids used | Number of oncospheres evaluated | Percentage of oncosphere activation |
| --- | --- | --- | --- | --- |
| MSHCF9431763 | Piura | 4 gravid | 50000 | 80% |
| FXVTM9655520 | Piura | 14 gravid | 35000 | 40% |
| MFGPF9711870 | Piura | 8 gravid | 50000 | 40% |
| EYCVM9790233 | Lima | 4 gravid | 72000 | 40% |
| AXPHM9818126 | Lima | 2 gravid | 35000 | 57% |

**Table B.** Distribution of cysticerci in the CNS of pigs according to hemisphere and anatomical location

| Brain hemisphere | All cysticerci (%) | Cyst anatomical location* | | | |
| --- | --- | --- | --- | --- | --- |
|  |  | Parenchymal (%) | Corticomeningeal (%) | Meningeal (%) | Cerebellum (%) |
| Left | 56 (42.7) | 18 (41.9) | 23 (44.2) | 3 (42.9) | – |
| Right | 69 (53.5) | 25 (58.1) | 29 (55.8) | 2 (28.6) | – |
| Midline | 3 (2.3) | 0 (0.0) | 0 (0.0) | 2 (28.6) | – |
| Cerebellum | 1 (0.8) | – | – | – | 1 (100.0) |
| Total | 129 (100.0) | 43 (100.0) | 52 (100.0) | 7 (100.0) | 1 (100.0 |
| *Cyst anatomical location was assessed in 103 cysticerci preserved in formalin | | | | | |

**Table C.** Detailed distribution of cysticerci (vesicular + degenerated) found in the carcasses of pigs according to oncosphere dose

| Organ | Total | | Oncosphere dose | | | | | |
| --- | --- | --- | --- | --- | --- | --- | --- | --- |
|  |  |  | 2500 | | 5000 | | 10000 | |
|  | n | % | n | % | n | % | n | % |
| Tongue | 232 | 6.52 | 5 | 1.54 | 50 | 3.69 | 177 | 9.42 |
| Head | 358 | 10.06 | 29 | 8.95 | 115 | 8.49 | 214 | 11.39 |
| Neck | 280 | 7.87 | 23 | 7.10 | 105 | 7.75 | 152 | 8.09 |
| Heart | 87 | 2.45 | 3 | 0.93 | 10 | 0.74 | 74 | 3.94 |
| Legs | 990 | 27.83 | 100 | 30.86 | 409 | 30.21 | 481 | 25.60 |
| Forelegs | 945 | 26.57 | 88 | 27.16 | 366 | 27.03 | 491 | 26.13 |
| Diaphragm | 385 | 10.82 | 43 | 13.27 | 175 | 12.92 | 167 | 8.89 |
| Sacrum | 280 | 7.87 | 33 | 10.19 | 124 | 9.16 | 123 | 6.55 |
| Total | 3557 | 100.00 | 324 | 100.00 | 1354 | 100.00 | 1879 | 100.00 |

**Table D.** Differences in the dynamics of circulating antigens and antibodies over time in experimentally infected pigs according to oncosphere dose–groups

| Oncosphere dose | Antigens | | Antibodies | |
| --- | --- | --- | --- | --- |
|  | Coefficient^†^  (95% CI) | *P* | Estimated mean^‡^ (95% CI) | *P* |
| 2500 | Ref. |  | Ref. |  |
| 5000 | 17.94 (9.13 - 26.74) | < 0.001 | -0.08 (-0.26 - 0.04) | 0.253 |
| 10000 | 18.83 (11.52 - 26.14) | < 0.001 | -0.17 (-0.52 - 0.07) | 0.228 |
| ^†^Coefficients were obtained by random–effects regression models for individual intercepts and robust standard errors; ^‡^ Estimated means for differences in the number of antibody bands between dose-groups were obtained by nonparametric kernel regression also using also time as covariate, and 200 bootstrap replications. | | | | |
